# Supplementary material for: Stochastic tuning of gene expression enables cellular adaptation in the absence of pre-existing regulatory circuitry
Source: eLife. 2018 Apr 5;7:e31867. doi: 10.7554/eLife.31867 (PMC5919758; doi:10.7554/eLife.31867)
Supplement: Supplementary file 3. — Shown are fitted values for the half-lives plus (in parentheses) the extent of a 95% confidence interval based on the model fit. All half-lives are given in minutes. [file elife-31867-supp3.pdf]

Overlap	SC+glu	6AU10
Top-Full	72.7 (42.4 – 256.0)	1172.8 (903.0 – 1673.1)
Top-Bottom	90.4 (60.2 – 181.5)	961.5 (799.5 – 1205.9)
Bottom-Full	101.4 (77.1 – 148.2)	370.5 (252.2 – 697.7)
