# Supplementary material for: Stochastic tuning of gene expression enables cellular adaptation in the absence of pre-existing regulatory circuitry
Source: eLife. 2018 Apr 5;7:e31867. doi: 10.7554/eLife.31867 (PMC5919758; doi:10.7554/eLife.31867)
Supplement: Supplementary file 4. — Numbers in parenthesis after mutation calls indicate the approximate fraction of the population containing the mutant allele. ‘ID’ is simply an identifier used to refer to each sample in the text. [file elife-31867-supp4.pdf]

ID	URA3 promoter	DHFR promoter	Media	Average genomic coverage	Average chromosome III coverage	Average URA3 insert coverage	URA3 sequence variants	Average DHFR insert coverage	DHFR sequence variants
C1	synprom	ADH1	6AU15 plate	14.4x	14.9x	15.3x	None	15.4x	None
C2	synprom	ADH1	6AU15 plate	10.9x	11.0x	11.8x	Synprom SNP (~30%), URA3 early stop (~30%)	10.4x	None
C3	synprom	ADH1	6AU 15 plate	25.2x	15.6x	31.3x	None	14.2x	None
C4	synprom	ADH1	6AU15 plate	13.2x	11.6x	10.6x	None	16.0x	None
L1	synprom	ADH1	Liquid 6AU5	30.6x	31.5x	33.7x	None	39.6x	None
L2	synprom	ADH1	Liquid 6AU5	6.8x	7.0x	8.5x	None	8.5x	None
L3	synprom	synprom	Liquid 6AU5	25.5x	27.4x	28.9x	None	25.9x	None
L4	synprom	synprom	Liquid 6AU5	8.7x	8.3x	7.9x	S->A in URA3-m Ruby linker (~40%)	8.7x	T->A in DHFR (~30%)
