# Supplementary material for: Stochastic tuning of gene expression enables cellular adaptation in the absence of pre-existing regulatory circuitry
Source: eLife. 2018 Apr 5;7:e31867. doi: 10.7554/eLife.31867 (PMC5919758; doi:10.7554/eLife.31867)
Supplement: Supplementary file 5. — Equal volumes of the sorted cells were plated in parallel on SC+glu and ura-/6AU15 plates, and then counted after 2–3 days (SC+glu) or 19–20 days (6AU). ‘Baseline’ refers to the fraction of cells expected to form colonies on 6AU15 plates in 19–20 days in unsorted populations (c.f. Figures 3–4 of the main text). [file elife-31867-supp5.pdf]

Promoter	Replicate 1			Replicate 2			Baseline
	SC+glu	*ura-/6A U15*	*Fraction forming colonies*	*SC+glu*	*ura-/6A U15*	*Fraction forming colonies*	
P_{RGI1}	66	0	<0.015	92	0	<0.011	0.0002
P_{HSP12}	189	14	0.074	98	5	0.051	0.15
synprom	256	0	<0.004	85	0	<0.012	0.0006
