# Supplementary material for: Stochastic tuning of gene expression enables cellular adaptation in the absence of pre-existing regulatory circuitry
Source: eLife. 2018 Apr 5;7:e31867. doi: 10.7554/eLife.31867 (PMC5919758; doi:10.7554/eLife.31867)
Supplement: Supplementary file 7. — End locations are given relative to the start codon of the gene in question. [file elife-31867-supp7.pdf]

Primer pair	Forward sequence	Reverse sequence	Target	5’ end	3’ end
URA3	ATGTCGAAAGCTACATATAAGGAACG	GGTGGTACGAACATCCAATGA	URA3	1	123
DHFR	ATGGTTCGACCATTGAACTGC	CACTGAAGAGGTTGTGGTCATTC	mDHFR	1	133
