# Supplementary material for: Stochastic tuning of gene expression enables cellular adaptation in the absence of pre-existing regulatory circuitry
Source: eLife. 2018 Apr 5;7:e31867. doi: 10.7554/eLife.31867 (PMC5919758; doi:10.7554/eLife.31867)
Supplement: Supplementary file 8. [file elife-31867-supp8.pdf]

Parameter	Description	Value
$\mathbf{p_{tunestep}}$	T mark addition probability	1
$\mathbf{n_{window}}$	Timesteps for fitness averaging	200
$\mathbf{p_{decay}}$	T mark decay probability	0.001
$\mathbf{p_{random}}$	Random T mark probability	0.1
$\mathbf{\mu_{max}}$	Maximum number of T marks	10
$\mathbf{p_{s\_mark}}$	S mark addition probability	0.005
$\mathbf{m_S}$	S mark effect size	0.01
